# High prevalence of non-O157 Shiga toxin-producing *Escherichia coli* in beef cattle detected by combining four selective agars

**DOI:** 10.1186/s12866-019-1582-8

**Published:** 2019-09-05

**Authors:** Ruyue Fan, Kun Shao, Xi Yang, Xiangning Bai, Shanshan Fu, Hui Sun, Yanmei Xu, Hong Wang, Qun Li, Bin Hu, Ji Zhang, Yanwen Xiong

**Affiliations:** 1 State Key Laboratory of Infectious Disease Prevention and Control, National Institute for Communicable Disease Control and Prevention, Chinese Center for Disease Control and Prevention, Changping, Beijing, China; 2 Shandong Center for Disease Control and Prevention, Jinan, Shandong Province China; 3 Zigong Center for Disease Control and Prevention, Zigong, Sichuan Province China; 4 mEpiLab, New Zealand Food Safety Science & Research Center, Institute of Veterinary, Animal and Biomedical Sciences, Massey University, Palmerston North, New Zealand

**Keywords:** Shiga toxin, *Escherichia coli*, O157:H7, Non-O157 STEC, Beef cattle, Culture medium

## Abstract

**Background:**

Shiga toxin-producing *Escherichia coli* (STEC) are emerging foodborne pathogens that are public health concern. Cattle have been identified as the major STEC reservoir. In the present study, we investigated the prevalence and characteristics of STEC strains in beef cattle from a commercial farm in Sichuan province, China.

**Results:**

Among 120 beef cattle fecal samples, *stx* genes were positive in 90% of samples, as assessed using TaqMan real-time PCR, and 87 (72.5%) samples were confirmed to yield at least one STEC isolate by culture using four selective agars, MacConkey, CHROMagar™ ECC, modified Rainbow® Agar O157, and CHROMagar™ STEC, from which 31, 32, 91, and 73 STEC strains were recovered, respectively. A total of 126 STEC isolates were selected and further characterized. Seventeen different O:H serotypes were identified, all of which belonged to the non-O157 serotypes. One *stx*_1_ subtype (*stx*_1a_) and three *stx*_2_ subtypes (*stx*_2a_, *stx*_2c_, and *stx*_2d_) were present among these isolates. The intimin encoding gene *eae*, and other adherence-associated genes (*iha*, *saa*, and *paa*) were present in 37, 125, 74, and 30 STEC isolates, respectively. Twenty-three isolates carried the virulence gene *subA*, and only one harbored both *cnf1* and *cnf2* genes. Three plasmid-origin virulence genes (*ehxA*, *espP*, and *katP*) were present in 111, 111, and 7 isolates, respectively. The 126 STEC isolates were divided into 49 pulsed-field gel electrophoresis (PFGE) patterns.

**Conclusions:**

Our study showed that the joint use of the selective MacConkey and modified Rainbow® Agar O157 agars increased the recovery frequency of non-O157 STEC strains in animal feces, which could be applied to other samples and in regular STEC surveillance. Moreover, the results revealed high genetic diversity of non-O157 STEC strains in beef cattle, some of which might have the potential to cause human diseases.

**Electronic supplementary material:**

The online version of this article (10.1186/s12866-019-1582-8) contains supplementary material, which is available to authorized users.

## Background

Shiga toxin-producing *Escherichia coli* (STEC) strains are significant foodborne zoonotic pathogens that are associated with illnesses ranging from mild diarrhea to hemorrhagic colitis (HC) and life-threatening hemolytic uremic syndrome (HUS) complications in humans [1]. Since the 1980s, more than 400 STEC serotypes have been reported worldwide, among which O157:H7 was the top causative serotype related to foodborne illnesses [2]. In recent years, sporadic cases or outbreaks caused by non-O157 STEC strains have been increasing [3–5]. Non-O157 STEC strains have been responsible for approximately 64% of STEC infections each year, particularly strains of several serogroups (O26, O45, O103, O111, O121, and O145, termed the “big six”), which generally possess the adhesin intimin (*eae* gene), a pathogenic marker for enterohemorrhagic *E. coli* (EHEC) and accounted for the majority of non-O157 STEC illnesses [2, 6, 7].

Shiga toxin (Stx), including Shiga toxin 1 (Stx1) and Shiga toxin 2 (Stx2) (encoded by *stx*_1_ and *stx*_2_ genes), are essential virulence factors in STEC strains [8]. Several Stx1 subtypes (Stx1a, Stx1c, and Stx1d) and Stx2 subtypes (Stx2a to Stx2h) have been identified in *E. coli* strains [9, 10]. The presence of *stx*_2a_ and/or *stx*_2c_ genes is more frequently related to severe clinical diseases [11]. In addition to Stx, a number of virulence factors are associated with pathogenicity. Intimin, encoded by the *eae* gene in the locus of enterocyte effacement (LEE), could induce attaching and effacing (A/E) lesions [12]. Strains containing a Shiga toxin gene together with the LEE island are classified as EHEC, which are associated with more severe clinical symptoms in humans [13]. Other adhesion-related genes such as *iha* (IrgA homolog adhesin), *efa1* (EHEC factor for adherence 1), *saa* (STEC autoagglutinating adhesin), and *paa* (porcine A/E associated protein) also play important roles in bacterial adhesion [14–16]. Cytotoxic necrotizing factors, encoded by the *cnf1* and *cnf2* genes, can impair the function of epithelial and immune cells. Subtilase (*subAB*) is an AB_5_ toxin that can lead to diseases including renal damage, hemolytic anemia, and HUS-related pathological features [1, 15]. The *astA* gene encodes a toxin that is structurally related to the heat-stable enterotoxin of enterotoxigenic *E. coli* [17]. In addition, the virulence factors carried on the pO157 plasmid are involved in STEC pathogenicity. For example, enterohemolysin (*ehxA*) can destroy mammalian cell membranes; serine protease (*espP*) and catalase-peroxidase (*katP*) contribute to the colonization of STEC strains in the human intestinal tract; and ToxB contributes to the adherence of O157:H7 to Caco-2 cells [18].

Although many domestic and wild animals can serve as a reservoir of STEC strains [19, 20], ruminants, especially cattle, have been recognized as the main reservoir and play an important role in the epidemiology of STEC infections [8, 21]. The transmission route includes ingestion of contaminated food or water, direct contact, or exposure to the environment [21]. The prevalence of STEC strains in cattle varied from 0.4 to 74.0% because of the differences in cattle categories, farm environments, and isolation methods [7, 22].

Being able to reliably detect STEC strains in different matrices could improve surveillance activities for emergent strains. To improve the recovery frequency of STEC strains, especially non-O157 STEC strains, from various samples, culture methods using selective chromogenic agars have been attempted [23–26]. Our previous investigation demonstrated that most of non-O157 STEC isolates recovered from diverse sources (animals, foodstuffs, and humans) in China were sensitive to tellurite ingredients, which resulted in poor growth on tellurite-amended agars. The recovery frequency of STEC strains from complex matrices could thus be improved by the combined use of less selective and highly selective agars [27]. In this study, we investigated the prevalence of STEC in the feces of beef cattle (collected from a commercial farm in China) using different selective chromogenic media. We further characterized these isolates by serotyping, *stx* subtyping, virulence gene profiling, and pulsed-field gel electrophoresis (PFGE) typing.

## Results

### Prevalence of *stx* genes in beef cattle fecal samples

Among the 120 fecal samples screened by TaqMan real-time PCR, 108 (90%) were positive for *stx*_1_ or *stx*_2_ or both (cycle threshold (Ct) values below 40), among which 80.8% of the samples were positive for both *stx*_1_ and *stx*_2_, while 9.2% (11 samples) were only *stx*_2_ positive (Additional file 1: Table S1).

### STEC isolates recovered from different selective chromogenic agars

Four different selective chromogenic agars, i.e. MAC (MacConkey), CH-ECC (CHROMagar™ ECC), RBA-NT (modified Rainbow® Agar O157 and CH-STEC (CHROMagar™ STEC), were used simultaneously to isolate STEC strains from all 120 samples. RBA-NT agar gave the highest positive culture rate of 59.2% (71/120), followed by CH-STEC agar (47.5%, 57/120). MAC and CH-ECC agars gave the same positive culture rate of 24.2% (29/120). In total, 72.5% (87/120) of the samples yielded one or more STEC isolate by the combined use of the four agars (Table 1). Notably, STEC isolates from two *stx*-PCR negative samples were recovered from both RBA-NT and CH-STEC agars. There was no significant correlation between the Ct values of *stx*-real-time PCR and the positive culture (Additional file 1: Table S1).

**Table 1:** STEC isolates recovered from different chromogenic agars

Agars	No. of samples with STEC isolates (%)	No. of STEC isolates	*stx*_1_ (%)	*stx*_2_ (%)	*stx*_1_ *+ stx*_2_ (%)
MAC	29 (24.2)	31	1 (3.2)	10 (32.3)	20 (64.5)
CH-ECC	29 (24.2)	32	1 (3.2)	10 (31.3)	21 (65.6)
RBA-NT	71 (59.2)	91	29 (31.9)	34 (37.4)	28 (30.8)
CH-STEC	57 (47.5)	73	22 (30.1)	31 (42.5)	20 (27.4)
Total	87 (72.5)	126^a^	31^a^	45^a^	50^a^

^a^Number of STEC isolates selected for further analyzing. MAC, MacConkey; CH-ECC, CHROMagar™ ECC, RBA-NT, modified Rainbow® Agar O157; CH-STEC, CHROMagar™ STEC

**Fig. 1:**
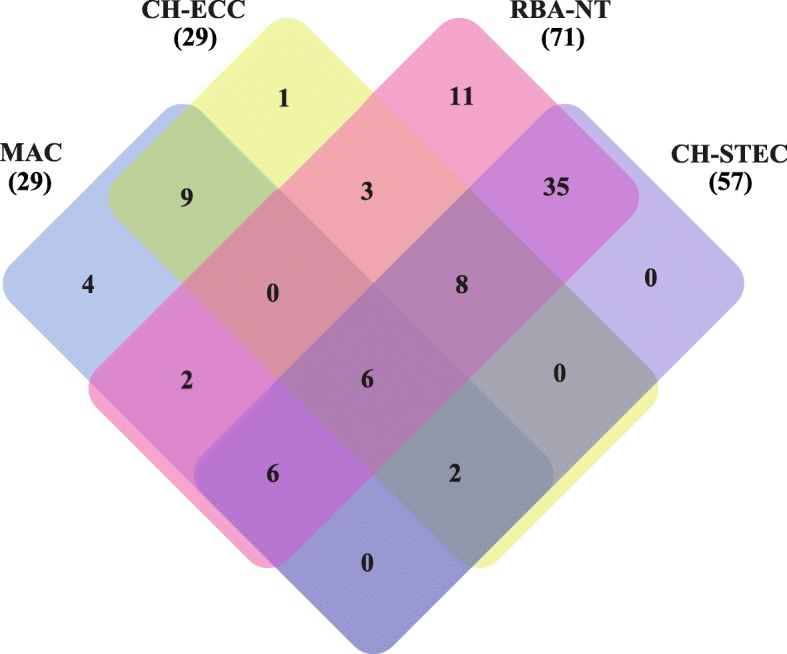
Venn diagram showing the numbers of common and unique samples that recovered STEC isolates on MAC, CH-ECC, RBA-NT, and CH-STEC agars. The number of culture-positive samples is listed in each of the diagram components. The total number of samples that recovered STEC isolates for each agar is given in parentheses

**Fig. 2:**
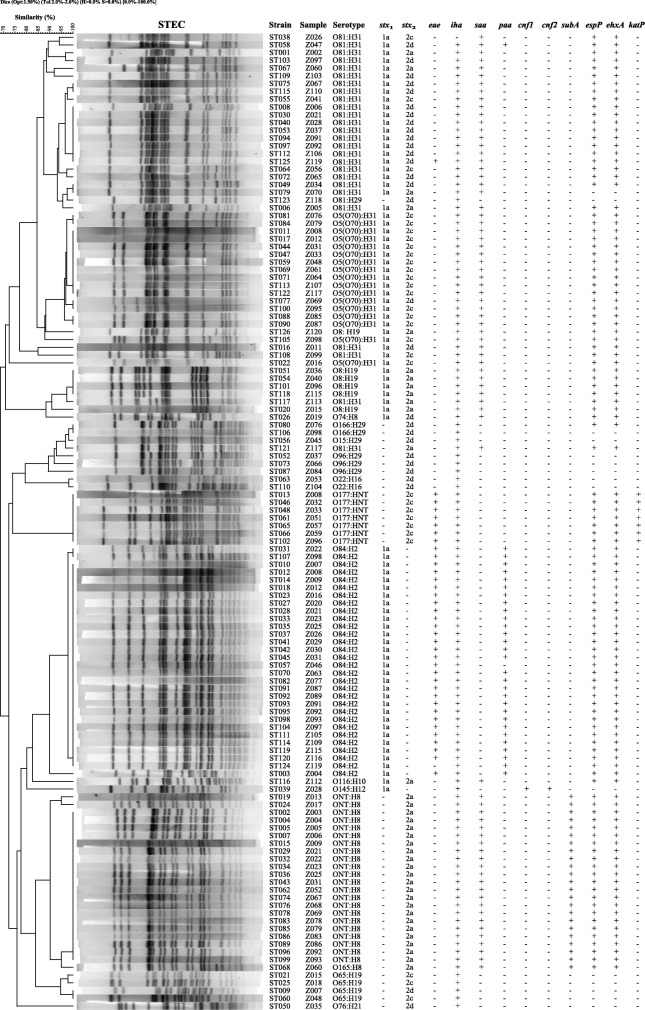
PFGE profiles of 126 non-O157 STEC isolates from beef cattle. The corresponding isolate names; samples names; serotypes; *stx*_1_ and *stx*_2_ subtypes; and the profiles of *eae*, *iha*, *saa*, *paa*, *cnf1*, *cnf2*, *subA*, *espP*, *ehxA*, and *katP* genes; are listed on the right

The performance of the four chromogenic agars was evaluated. Only six samples yielded STEC isolates on all four agars. Although RBA-NT and CH-STEC agars gave higher culture positive rates, the combined use of MAC and RBA-NT agars could cover almost all strains cultured from the other two agars (except for one sample) (Fig. 1). In total, 31, 32, 91, and 73 *stx*-positive isolates were recovered from 120 samples on MAC, CH-ECC, RBA-NT, and CH-STEC agars, respectively. Twenty (64.5%) and 21 (65.6%) strains that were positive for the *stx*_1_ *+ stx*_2_ genes were found on MAC and CH-ECC agars, respectively. Among the isolates recovered on RBA-NT agar, 29 *stx*_1_-positive and 34 *stx*_2_-positive strains were tested, and 28 tested positive for both *stx*_1_ and *stx*_2_ genes. Of the 73 isolates found on CH-STEC agar, 22 and 31 tested positive for the *stx*_1_ and *stx*_2_ genes, respectively, and 20 tested positive for both genes. With the exception of the different *stx* types/subtypes present in the same sample, only one representative STEC isolate from each sample was kept. Finally, one isolate carrying the *stx*_1_, *stx*_2_, or *stx*_1_ *+ stx*_2_ gene was chosen from 53 samples each; two isolates per sample were chosen from 29 samples; and three isolates per sample were chosen from five samples. A total of 126 *stx*-positive isolates were all confirmed to be *E. coli* and were selected for further characterization (Table 1 and Fig. 2).

### Serogroups and serotypes

Fifteen different O serogroups and nine different H types were identified among the 126 STEC isolates, which belonged to 17 different serotypes, including O5(O70):H31, O8:H19, O15:H29, O22:H16, O65:H19, O74:H8, O76:H21, O81:H31, O81:H29, O84:H2, O96:H29, O116:H10, O145:H12, O165:H8, O166:H29, O177:HNT, and ONT:H8. The most common serotype was O84:H2 (23.81%), followed by O81:H31 (20.63%), ONT:H8 (17.46%), and O5(O70):H31 (13.49%). Twenty-two and seven isolates were untypable for the O and H antigens, respectively (Table 2).

**Table 2:** Serotypes, *stx* subtypes and virulence genes of 126 beef cattle STEC isolates

Serotype	No. of isolates	*stx* subtypes		Intimin gene	Adherence-related genes			Other virulence-associated genes			Plasmid genes		
		*stx*_1_	*stx*_2_	*eae*	*iha*	*saa*	*paa*	*cnf1*	*cnf2*	*subA*	*ehxA*	*espP*	*katP*
O5(O70):H31 ^a^	16	*stx*_1a_	*stx*_2c_	–	+	+	–	–	–	–	+	+	–
O5(O70):H31 ^a^	1	*stx*_1a_	*stx*_2d_	–	+	+	–	–	–	–	+	+	–
O8: H19	6	*stx*_1a_	*stx*_2a_	–	+	+	–	–	–	–	+	+	–
O15:H29	1		*stx*_2d_	–	+	–	–	–	–	–	–	–	–
O22:H16	2		*stx*_2d_	–	+	–	–	–	–	–	–	–	–
O65:H19	1		*stx*_2d_	–	+	–	–	–	–	–	–	–	–
O65:H19	3		*stx*_2c_	–	+	–	–	–	–	–	–	–	–
O74:H8	1	*stx*_1a_	*stx*_2d_	–	+	+	–	–	–	–	+	+	–
O76:H21	1		*stx*_2d_	–	+	–	–	–	–	–	–	–	–
O81:H29	1		*stx*_2d_	–	+	–	–	–	–	–	–	–	–
O81:H31	4	*stx*_1a_	*stx*_2a_	–	+	+	–	–	–	–	+	+	–
O81:H31	1	*stx*_1a_	*stx*_2a_	–	+	+	–	–	–	–	–	–	–
O81:H31	1		*stx*_2a_	–	+	+	–	–	–	–	+	+	–
O81:H31	3	*stx*_1a_	*stx*_2c_	–	+	+	–	–	–	–	+	+	–
O81:H31	1	*stx*_1a_	*stx*_2c_	–	–	+	–	–	–	–	+	+	–
O81:H31	14	*stx*_1a_	*stx*_2d_	–	+	+	–	–	–	–	+	+	–
O81:H31	1	*stx*_1a_	*stx*_2d_	+	+	+	–	–	–	–	+	+	–
O81:H31	1	*stx*_1a_	*stx*_2d_	–	+	+	+	–	–	–	+	+	–
O84:H2	28	*stx*_1a_		+	+	–	+	–	–	–	+	+	–
O84:H2	1	*stx*_1a_		–	+	–	+	–	–	–	+	+	–
O84:H2	1	*stx*_1a_		+	+	–	–	–	–	–	+	+	–
O96:H29	3		*stx*_2d_	–	+	–	–	–	–	–	–	–	–
O116:H10	1	*stx*_1a_	*stx*_2a_	–	+	+	–	–	–	–	+	+	–
O145:H12	1	*stx*_1a_		–	+	–	–	+	+	–	–	–	–
O165:H8	1		*stx*_2a_	–	+	+	–	–	–	+	+	+	–
O166:H29	1		*stx*_2d_	–	+	–	–	–	–	–	+	+	–
O166:H29	1		*stx*_2d_	–	+	–	–	–	–	–	–	–	–
O177: HNT	7		*stx*_2c_	+	+	–	–	–	–	–	+	+	+
ONT:H8	22		*stx*_2a_	–	+	+	–	–	–	+	+	+	–
Total	126	1 ^b^	3 ^b^	37	125	74	30	1	1	23	111	111	7

^a^, These isolates were agglutinated with both O5 and O70 antisera

^b^, Number of *stx*_1_ or *stx*_2_ subtypes identified

### Subtypes of *stx*

Of the 126 isolates, 31 (24.6%) were positive for *stx*_1_ only, 45 (35.7%) were positive for *stx*_2_ only, and 50 (39.7%) were positive for both *stx*_1_ and *stx*_2_. Only the *stx*_1a_ subtype was identified in the 81 *stx*_1_-positive STEC isolates. Among 95 *stx*_2_-positive isolates, three subtypes, i.e. *stx*_2a_, *stx*_2c_, and *stx*_2d_, were identified in 36, 30, and 29 isolates, respectively (Table 2).

### Presence of virulence genes

All STEC isolates carried at least one of the virulence-related genes tested. Thirty-seven isolates were *eae* positive, which belonged to only three serotypes (O81:H31, O84:H2, and O177:HNT). Among the putative adhesin genes (*iha*, *efa1*, *saa*, and *paa*) screened, *iha* (99.2%) was the most prevalent, followed by *saa* (58.7%) and *paa* (23.8%); however, *efa1* was not detected in any isolate. Among the virulence-associated genes (*cnf1*, *cnf2*, *astA*, and *subA*) tested, 23 (18.3%) isolates were positive for *subA*, and only one isolate contained both *cnf1* and *cnf2*. The *astA* gene was absent in all STEC isolates. Three plasmid-related virulence genes (*ehxA*, *espP*, and *katP*) were present in 111, 111 and 7 isolates, respectively. Notably, *ehxA-*positive isolates also carried *espP*, and the seven *katP*-positive isolates harbored both *ehxA* and *espP*. None of the 126 isolates were positive for *toxB* (Table 2).

### Pulsed field gel electrophoresis (PFGE)

Genomic DNA from all 126 isolates was digested using *Xba* I and separated using PFGE to investigate their genetic relationships. An UPGMA (unweighted pair group method with arithmetic mean) dendrogram showed that the STEC isolates were genetically diverse, with nodes linking single isolates or groups of isolates at less than 80% similarity. In total, 49 different PFGE patterns were generated among the 126 STEC isolates. There were two predominant PFGE patterns, which contained 27 and 20 isolates each. Most isolates in the same PFGE pattern tended to have the same serotype, *stx* subtypes, and virulence gene profiles. All isolates recovered from the same sample showed different PFGE patterns, serotypes, or virulence gene profiles to each other (Fig. 2).

## Discussion

Cattle have been identified as dominant reservoirs of STEC without showing any clinical signs themselves, and the consumption of food or water contaminated with bovine feces is often linked to STEC infection [11]. The prevalence of STEC in cattle has been reported recently in the United States, France, Australia, Japan, Brazil, and other countries [1–3, 13, 28–30]. According to previous reports, the prevalence rates of O157 and non-O157 STEC strains ranged from 22 to 62.7% and 2.1 to 70.1%, respectively, in different categories of cattle [22, 31]. In recent years, molecular techniques have been developed to target STEC strains. A TaqMan real-time PCR method was adopted in this study to screen for the presence of STEC strains. A high *stx-*positive rate (90%) was obtained in beef cattle fecal samples after enrichment, showing the high sensitivity of the real-time PCR method [32]. The isolation rate in this study was 72.5% by culture, indicating a high prevalence of STEC in these beef cattle. The difference of STEC prevalence rates might depend on the various cattle species in certain geographic areas and different detection methods [22]. The failure to recover isolates from a fraction of *stx*-positive samples in this study indicated that the *stx* gene might have been amplified from non-*E. coli* bacteria, *stx*-phages, or free DNA molecules in the background flora. Another possibility is perturbation of background microflora, or low levels of STEC strains in the *stx*-positive samples. Notably, two *stx*-negative samples yielded STEC strains using the culture method, indicating that the presence of STEC strains in cattle fecal samples may be underestimated if only *stx*-positive samples are subjected to culture. The disagreement between culture- and PCR-based methods to detect STEC strains in beef fecal samples was also observed in a previous report [33]. One of the reasons for the misidentification of culture-positive samples by real-time PCR was likely explained by the inhibitors in samples inducing an increase of Ct values [34].

Currently, there is no single or combination of multiple selective agars capable of identifying all STEC serogroups [35]. As suggested by our previous study, using inclusive agars or less selective agars in combination with highly selective agars would increase the probability of recovering STEC strains from complex matrices [27]. By combining two inclusive and less selective agars (MAC and CH-ECC) and two highly selective agars (RBA-NT and CH-STEC), a culture positive rate of 78.7% (85/108) from the *stx*-positive samples was obtained, which was higher than those reported in our previous investigation in yak (61.6%) [36], pig (24.4%) [37], and pika (36.2%) [19]. In general, one to three STEC strains were obtained using the four agars for each sample. One strain was predominantly obtained on MAC or CH-ECC agar, which mainly carried *stx*_1_ *+ stx*_2_ genes. One or two strains were predominantly obtained on RBA-NT and CH-STEC agars; however, there was no obvious *stx* subtype difference between the two agars (Table 1). Notably, the combination of MAC and RBA-NT could cover almost all the culture positive samples (except for one sample) (Fig. 1). Both CH-STEC and RBA-NT use potassium tellurite as a selective additive to isolate specific bacteria; however, STEC isolates display a great variation in tellurite resistance [27, 38]. Our study indicated that MAC may cover the shortage of tellurite-amended agars, and that the combined using MAC and RBA-NT agars could increase the recovery frequency of non-O157 STEC from animal fecal samples.

O:H serotyping of STEC strains has been used widely to identify a strain’s potential to cause severe diseases. O157:H7 is the most well known STEC serotype that causes infections and outbreaks worldwide; however, other serotypes might show regional variations. In particular, STEC strains that possess both the Stx toxin and intimin are the causative agents of severe clinical outcomes, and are also classified as EHEC [13]. Five O groups (O157, O26, O103, O111, and O145), are known as the “big five” EHEC in the European Union, while six O types (O26, O45, O103, O111, O121, and O145), or the “big six”, have been recognized as being responsible for most of the clinical non-O157 STEC infections in the United States [39]. The O157:H7 serotype was not identified in the present study, as was the case with our previous surveys in yak, pig, pika, and raw meat [19, 36, 37, 40], implying a low prevalence of O157 STEC in China. Another possible reason for failure to isolate an O157 STEC might be the method used. Isolation of O157 STEC strains often requires more targeted methods, and the use of immunomagnetic separation (IMS) may improve the isolation sensitivity of O157 strains. However, one of the “big six” serotypes, O145:H12, was identified in this study. Several serogroups of bovine-origin such as O8, O15, O22, O84, O165, and O116 were also detected, some of which are associated with human diseases [1, 3, 31, 41].

STEC is characterized by the production of Stx1, Stx2, or both, and several Stx1/Stx2 subtypes have been described in *E. coli* [10]. A novel subtype of Stx1e was identified in *Enterobacter cloacae* [42]. Recently, we identified a novel Stx2 subtype, Stx2h, in *E. coli* strains from wild marmots in the Qinghai-Tibetan plateau, China [9]. Stx subtypes differ dramatically in their pathogenic potency. The Stx1a, Stx2a, Stx2c, and Stx2d subtypes are commonly reported as associated with HC and HUS [39]. In this study, all *stx*_1_-positive strains in beef cattle carried the *stx*_1a_ subtype, which was consistent with previous reports [3, 8], while the most prevalent *stx*_2_ subtypes were *stx*_2a_ subtypes (53.3%). Three *stx* subtype combinations, i.e. *stx*_1a_ + *stx*_2a_, *stx*_1a_ + *stx*_2c_, and *stx*_1a_ + *stx*_2d_ were detected in 39.7% of the isolates, suggesting that some STEC isolates from cattle have a high pathogenic potential.

To further evaluate the potential virulence of STEC isolates from the cattle, virulence factor genes were tested. Intimin, encoded by the *eae* gene, plays an important role in bacterial colonization, and several studies have documented that STEC strains carrying the *eae* gene, which are also identified as EHEC strains, are highly associated with severe human diseases and outbreaks [43, 44]. The *eae*-positive rate among the 126 STEC isolates in this study was 29.4%, 29 *eae*-positive isolates carried *stx*_1a_, seven harbored *stx*_2c_, and one possessed both *stx*_1a_ and *stx*_2d_, which was similar to previous reports [8, 30], implying their high pathogenic potential. Other adherence-related genes, *iha*, *saa* and *paa*, were present at varying frequencies among the isolates, which may have involved cattle colonization by *eae*-negative non-O157 STEC strains [44]. Additionally, co-existence of *ehxA* and *espP*, two plasmid-origin virulence factors, were observed, which contrasted with reports of non-O157 STEC isolates from other sources [45]. The subtilase toxin encoded by *subA* gene was described in STEC O113:H21, which was related to an outbreak of HUS [46]. In total, 23 isolates from the cattle fecal samples harbored *subA* genes; however, these STEC strains belonged to the O165 and untypable O serogroups.

PFGE is often used as the gold standard molecular typing method for intestinal pathogens [47]. For those two or three colonies selected from the same sample with different phenotypic or genetic properties, different PFGE patterns, serotypes, and virulence gene profiles were observed, which indicated that beef cattle were colonized by different STEC strains. Some isolates from different samples showed identical PFGE patterns, serotypes, and virulence gene profiles, suggesting that multiple isolates from different beef cattle may belong to the same STEC clone and cross contamination might have occurred on the farm.

## Conclusions

A considerable number of non-O157 STEC strains were isolated from beef cattle feces in a farm in China. Based on their serotypes, *stx* subtypes, and the presence of virulence genes, some non-O157 STEC isolates from beef cattle may have the potential to cause human diseases. A combination of selective MAC and RBA-NT media could increase the recovery frequency of non-O157 STEC during regular animal surveillance.

## Methods

### Sample collection and enrichment

A total of 120 beef cattle fecal samples were collected from five different pens in a commercial beef cattle farm in Zigong city, Sichuan province, China, in May 2017. All fresh fecal samples were stored separately in 2 ml sterile tubes containing Luria-Bertani medium (LB, Land Bridge, Beijing, China) with 30% glycerol, and then transported immediately to the laboratory in the National Institute for Communicable Disease Control and Prevention, China CDC, in ice bags under cold conditions. After arrival in the laboratory, the sample was frozen at − 80 °C until culture. Each fecal sample was homogenized in 5 ml of *E. coli* broth (EC broth, Land Bridge, Beijing, China) after thawing, and then incubated at 37 °C for 18 to 24 h with agitation.

### DNA extraction and *stx* screening by TaqMan real-time PCR

A portion (1.5 ml) of each enrichment culture was transferred to a new tube for DNA extraction and centrifugation. Briefly, 150 μl of rapid lysis buffer (100 mM NaCl, 1 mM EDTA [pH 9.0], 10 mM Tris-HCl [pH 8.3], 1% Triton X-100) were added to 1.5 ml of each centrifuged pellet. The mixture was boiled for 10 min, followed by centrifuged at 13,000×*g* for 10 min, and the supernatant containing the DNA was used as a template in real-time PCR assays. The primers and probes set for *stx*_1_/*stx*_2_ detection was prepared as previously described [32].

### Isolation of STEC strains

Approximately 10 μl of each enrichment culture was streaked directly onto CHROMagar™ ECC agar (CH-ECC, CHROMagar, Paris, France), MacConkey agar (MAC, Land Bridge, Beijing, China), and Rainbow® Agar O157 (RBA, Biolog Inc., Hayward, CA, USA), supplemented with 10 μg/ml novobiocin and 0.8 μg/ml potassium tellurite (modified, RBA-NT), and CHROMagar™ STEC agar (CH-STEC, CHROMagar, Paris, France), respectively. After overnight incubation at 37 °C, ten presumptive colonies on each plate were picked and subjected to colony PCR to detect the *stx*_1_ and *stx*_2_ genes according to the method revealed in a previous study [19], including green-blue or colorless colonies on CH-ECC agar; pink or colorless colonies on MAC agar; purple, grey, or mauve colonies on RBA-NT agar; and mauve colonies on CH-STEC agar. The *stx*-positive colonies were then plated onto LB agar and incubated at 37 °C overnight to obtain a single colony for further identification. Only one STEC isolate from each sample was chosen for further characterization if only identical stx types/subtypes were present on the four different agars. Isolates on inclusive agars (MAC and CH-ECC agars) were selected as a priority, while other isolates with same stx types/subtypes were eliminated.

### Biochemical test and serotyping of STEC isolates

All *stx*-positive isolates were confirmed biochemically as *E. coli* using API® 20E biochemical test strips (bioMérieux, Lyon, France). The O serogroup of each isolate was preliminary screened using a PCR method with O antigen specific primers [48] and confirmed by using all O1-O188 *E. coli* antisera (SSI Diagnostica, Hillerød, Denmark). The entire coding sequence of the *fliC* gene was amplified by PCR, and then sequenced to determine the H type, as described previously [19].

### Identification of virulence and adherence factor genes

The presence of the intimin-encoding gene (*eae*), putative adhesin genes (*iha*, *efa1*, *saa* and *paa*), virulence-associated genes (*cnf1*, *cnf2*, *astA*, and *subA*), and the large heterologous virulence plasmid genes (*ehxA*, *katP*, *espP*, and *toxB*) in all STEC isolates were detected as previously described [36].

### Subtyping of *stx*

The *stx*_1_ and *stx*_2_ subtypes were determined using a PCR-based subtyping method [10]. The complete *stx*_1_ and/or *stx*_2_ genes of certain STEC isolates were amplified and sequenced to verify the PCR-based subtyping results.

### Pulsed-field gel electrophoresis (PFGE)

PFGE was performed according to the STEC subtyping protocol from PulseNet, USA (https://www.cdc.gov/pulsenet/pdf/ecoli-shigella-salmonella-pfge-protocol-508c.pdf). Briefly, the genomic DNA was digested for 2 h with 45 U of *Xba*I (Takara, Dalian, China) at 37 °C. The digested samples were placed on 1% SeaKem Gold agarose and electrophoresis was carried out at 6.0 V/cm for 18 h with an initial switch time value of 6.8 s and final switch time of 35.5 s. Images were captured using the Gel Doc™ XR+ System (Bio-Rad, Hercules, CA, USA). PFGE patterns were analyzed and a UPGMA dendrogram was constructed using the BioNumerics software (Applied Maths, Belgium).

## Additional file

Additional file 1: **Table S1.** Prevalence of *stx* and STEC in beef cattle feces. (PDF 39 kb)

## Data Availability

All data generated or analyzed during this study are included in this published article and its supplementary information files.

## Abbreviations

A/E: Attaching and Effacing
CH-ECC: CHROMagar™ ECC
CH-STEC: CHROMagar™ STEC
EHEC: Enterohemorrhagic *E. coli*
HC: Hemorrhagic colitis
HUS: Hemolytic uremic syndrome
IMS: Immunomagnetic separation
LEE: Locus of enterocyte effacement
MAC: MacConkey
PFGE: Pulsed-Field Gel Electrophoresis
RBA-NT: modified Rainbow® Agar O157
STEC: Shiga toxin-producing *Escherichia coli*
Stx: Shiga toxin
Stx1: Shiga toxin 1
Stx2: Shiga toxin 2
UPGMA: Unweighted Pair Group Method with Arithmetic mean
