# Supplementary material for: High prevalence of non-O157 Shiga toxin-producing Escherichia coli in beef cattle detected by combining four selective agars
Source: BMC Microbiol. 2019 Sep 5;19:213. doi: 10.1186/s12866-019-1582-8 (PMC6728992; doi:10.1186/s12866-019-1582-8)
Supplement: Supplementary file 1 — Table S1. Prevalence of stx and STEC in beef cattle feces. (PDF 39 kb) [file 12866_2019_1582_MOESM1_ESM.pdf]

Table S1. Prevalence of *stx* and STEC in beef cattle feces

Sample	Real-time PCR (Ct)		Chromogenic agars			
	stx$_1$	*stx*$_2$	MAC	CH-ECC	RBA-NT	CH-STEC
Z001	35.72	34.94	-	-	-	-
Z002	25.80	25.07	-	-	+	-
Z003	23.09	22.53	-	-	+	-
Z004	26.30	25.38	-	-	+	+
Z005	26.87	26.09	-	-	+	+
Z006	24.43	22.58	-	+	+	+
Z007	20.64	18.91	+	-	+	+
Z008	25.96	25.87	+	-	+	+
Z009	28.25	28.31	-	-	+	+
Z010	36.06	34.23	-	-	-	-
Z011	29.92	29.63	+	-	+	+
Z012	23.06	25.05	+	+	+	+
Z013	-	21.05	-	-	+	-
Z014	-	-	-	-	-	-
Z015	24.31	22.05	-	+	+	+
Z016	27.34	26.79	-	+	+	-
Z017	36.47	35.30	-	-	+	-
Z018	31.09	30.01	-	-	+	-
Z019	23.45	22.52	+	-	-	-
Z020	31.15	27.09	-	-	+	+
Z021	30.51	26.32	-	-	+	+
Z022	27.79	26.08	-	-	+	+
Z023	22.38	21.59	-	-	+	+
Z024	27.78	31.84	-	-	-	-
Z025	19.95	21.20	-	-	+	-
Z026	23.99	23.99	-	-	+	+
Z027	25.75	20.67	-	-	-	-
Z028	21.63	22.22	+	-	+	-
Z029	24.98	24.33	-	-	+	+
Z030	23.75	23.47	-	-	+	+
Z031	20.00	18.59	-	+	+	+
Z032	23.17	20.82	-	-	+	+
Z033	22.44	22.17	+	+	+	+

Z034	24.50	25.84	-	-	+	-
Z035	-	18.94	+	+	-	+
Z036	22.82	22.93	-	+	-	-
Z037	23.19	23.51	+	+	+	+
Z038	-	-	-	-	-	-
Z039	-	-	-	-	-	-
Z040	20.89	21.59	+	+	+	+
Z041	30.87	32.42	-	-	+	-
Z042	-	-	-	-	-	-
Z043	-	-	-	-	-	-
Z044	25.17	25.10	-	-	-	-
Z045	19.39	19.25	+	-	-	-
Z046	29.24	32.92	-	-	+	+
Z047	24.09	26.58	-	+	+	+
Z048	17.64	18.68	+	+	+	+
Z049	-	-	-	-	-	-
Z050	-	23.07	-	-	-	-
Z051	35.28	32.02	-	-	+	+
Z052	-	29.50	-	-	+	+
Z053	-	21.65	+	+	-	-
Z054	34.15	34.98	-	-	-	-
Z055	28.44	29.10	-	-	-	-
Z056	28.82	25.86	-	-	+	-
Z057	-	31.29	-	-	+	+
Z058	23.86	24.03	-	-	-	-
Z059	-	25.96	-	-	+	+
Z060	20.87	21.54	-	+	+	+
Z061	21.48	22.28	+	+	-	-
Z062	27.13	27.25	-	-	-	-
Z063*	-	-	-	-	+	+
Z064	23.42	24.56	+	+	-	-
Z065	25.59	25.96	-	-	+	+
Z066	-	22.84	+	+	-	-
Z067	19.80	22.07	-	-	+	+
Z068	28.13	24.78	-	+	+	+
Z069	26.77	20.07	-	+	+	+

Z070	30.82	31.31	-	-	+	+
Z071	36.06	39.29	-	-	-	-
Z072	26.02	27.00	-	-	-	-
Z073	-	-	-	-	-	-
Z074	22.70	22.33	-	-	-	-
Z075	27.28	28.26	-	-	-	-
Z076	22.55	23.81	+	-	+	-
Z077	26.79	27.44	-	-	+	+
Z078	23.05	23.43	-	-	+	+
Z079	24.85	25.80	+	-	+	+
Z080	-	-	-	-	-	-
Z081	34.94	35.08	-	-	-	-
Z082	25.84	25.75	-	-	-	-
Z083*	-	-	-	-	+	+
Z084	-	21.62	+	+	-	-
Z085	24.06	23.81	-	+	+	-
Z086	32.94	26.78	-	-	+	+
Z087	24.82	25.77	+	-	+	+
Z088	36.57	37.60	-	-	-	-
Z089	28.16	28.55	-	-	+	+
Z090	31.87	30.31	-	-	-	-
Z091	25.65	28.38	-	-	+	+
Z092	23.56	24.46	-	-	+	+
Z093	25.06	26.12	-	-	+	+
Z094	27.48	28.83	-	-	-	-
Z095	23.97	25.13	+	-	-	-
Z096	18.55	18.29	+	+	+	+
Z097	22.18	22.61	+	-	+	+
Z098	22.94	24.06	+	+	-	+
Z099	23.30	24.08	-	+	+	+
Z100	-	26.43	-	-	-	-
Z101	-	-	-	-	-	-
Z102	-	-	-	-	-	-
Z103	32.59	28.90	-	-	+	+
Z104	-	22.14	+	+	-	-
Z105	29.89	36.74	-	-	+	+

Z106	29.65	30.32	-	-	+	+
Z107	18.48	19.91	+	+	-	-
Z108	27.10	27.02	-	-	-	-
Z109	32.96	33.05	-	-	+	+
Z110	23.53	23.28	-	-	+	+
Z111	21.22	22.89	-	-	-	-
Z112	24.51	23.71	+	-	-	-
Z113	20.75	21.63	+	+	-	-
Z114	26.23	27.31	-	-	-	-
Z115	22.81	23.12	-	+	+	-
Z116	35.85	36.20	-	-	+	-
Z117	21.05	22.11	+	+	-	-
Z118	34.58	32.84	-	-	+	+
Z119	22.58	24.18	-	-	+	+
Z120	23.48	24.55	-	-	+	-
**Total**	**97**	**108**	**29**	**29**	**71**	**57**

MAC, MacConkey agar; CH-ECC, CHROMagar™ ECC agar; RBA-NT, Rainbow® Agar O157; CH-STEC, CHROMagar™ STEC agar.

*The two *stx*-negative samples yielded STEC isolates.
